# Efficient Estimation of Marker Effects in Plant Breeding

**DOI:** 10.1534/g3.119.400728

**Published:** 2019-09-19

**Authors:** Alencar Xavier

**Affiliations:** * Corteva Agrisciences, 8305 NW 62nd Ave. Johnston IA, and; † Purdue University, 915 W State St. West Lafayette IN

**Keywords:** Mixed model, Laplace prior, Single-stage, Gauss-Seidel, Predictability, Elapsed time, Genomic Prediction, GenPred, Shared Data Resources

## Abstract

The evaluation of prediction machines is an important step for a successful implementation of genomic-enabled selection in plant breeding. Computation time and predictive ability constitute key metrics to determine the methodology utilized for the consolidation of genomic prediction pipeline. This study introduces two methods designed to couple high prediction accuracy with efficient computational performance: 1) a non-MCMC method to estimate marker effects with a Laplace prior; and 2) an iterative framework that allows solving whole-genome regression within mixed models with replicated observations in a single-stage. The investigation provides insights on predictive ability and marker effect estimates. Various genomic prediction techniques are compared based on cross-validation, assessing predictions across and within family. Properties of quantitative trait loci detection and single-stage method were evaluated on simulated plot-level data from unbalanced data structures. Estimation of marker effects by the new model is compared to a genome-wide association analysis and whole-genome regression methods. The single-stage approach is compared to a GBLUP fitted via restricted maximum likelihood, and a two-stages approaches where genetic values fit a whole-genome regression. The proposed framework provided high computational efficiency, robust prediction across datasets, and accurate estimation of marker effects.

Genome-wide markers are utilized in plant and animal breeding to capture quantitative trait loci (QTL) and relationship among individuals for prediction and selection (Meuwissen *et al.* 2001, Habier *et al.* 2007, VanRaden 2008). Most individuals in the plant breeding pipeline are genotyped, whereas in animal breeding genomic information enhances the pedigree-based relationship (Henryon *et al.* 2014). With the ever increasing volume of genotypic and phenotypic data, various statistical methods have been developed to handle large datasets, enabling better use of genomic information for more accurate selection and better allocation of resources (Heslot *et al.* 2012).

Evaluating the predictive performance of these various methodologies has become an important step for a successful implementation of genomic-enabled selection (de los Campos *et al.* 2013, Heslot *et al.* 2015), since the prediction method utilized to generate breeding values may have major impact on the short-term genetic gain, as well as long-term changes on the germplasm (Daetwyler *et al.* 2015, Hickey *et al.* 2017).

Genomic predictions models are used to estimate breeding values of observed individuals and to predict breeding values of unobserved individuals in early-generations. Accuracy is the most important criterion to define which technique will be used to generate the breeding values. Besides accuracy, the computational efficiency has also become a key component of prediction pipelines due to the growing number of genotyped individuals, observations per individuals, traits, and genotyping density (Georges *et al.* 2018). Hence the method of choice must have two desirable features: computational feasibility and accurate prediction across various scenarios (VanRaden 2008, Misztal and Legarra 2017).

In plant breeding, the calibration of such models are typically done in two steps: 1) Estimate the genetic values from phenotypes of replicated trials; 2) Calibrate marker effects upon the genetic values to estimate the breeding values and enable prediction. This approach is referred to as a ”two-stage” approach. However, single-stage analysis can benefit genomic evaluation by jointly modeling genotypes and replicated phenotypes (Liu *et al.* 2014).

Few studies have attempted to estimate marker effects directly from replicated trials. Taskinen *et al.* (2017) proposed using pedigree information of ungenotyped individuals for imputation and subsequent estimation of marker effects. Da *et al.* (2014) provided two frameworks to fit genomic models to estimate variance components and marker effects, one approach suitable for large number of observations and another for large number of markers, but not for both. However, such methods often translate into poor computational performance or convergence issues (Misztal 2016).

Fernando *et al.* (2014, 2016) provided a framework where marker effects can be estimated from whole-genome regression (WGR) methods via Markov chain Monte Carlo (MCMC), enabling a broader range of prior assumptions for the distribution of marker effects that can provide predictive advantages in single-stage approaches (Zhou *et al.* 2018).

Flexible models that enable the estimation of marker effects among other parameters are commonly based on MCMC method (Fernando *et al.* 2014), but these techniques can be computationally prohibitive at times (Wang *et al.* 2015) and must be replaced by Gauss–Seidel iterations (Garrick *et al.* 2014).

This study proposes an efficient non-MCMC solver for WGR and mixed models based on conditioning and iterative updates. The idea is to develop a single-stage solver by jointly iterating the steps of the multi-stage analysis. Predictive ability and computing time of the proposed framework are evaluated through simulations and cross-validation on real data, comparing it to other methods.

## Statistical models

Iterative conditional modeling enables solving complex models without the computationally demanding operations (Graser *et al.* 1987, Thompson and Shaw 1992, Misztal and Legarra 2017). In these methods, conditional expectations are used to efficiently estimate variance components, fixed effects, breeding values, and marker effects (Da *et al.* 2014, Liu *et al.* 2014, Fernando *et al.* 2014, Taskinen *et al.* 2017). Two statistical approaches are introduced in this section. First, an iterative algorithm for WGR that speeds up the marker calibration. Second, a framework to enables solving WGR into a model with replicated observations using a specific type of conditioning.

## Whole-genome model

This section describes the implementation of the fast Laplace model (FLM), an iterative method to fit a WGR using a Laplace prior. Laplace priors are popular in genetic analysis for QTL detection and genomic prediction (Xu 2007, Xu 2010, Cai *et al.* 2011, Legarra *et al.* 2011).

The implementation below is based on iterative conditional expectation (ICE) estimates of regression coefficients alongside their associated parameters, updating one parameter at a time (Meuwissen *et al.* 2009). This type of algorithm is commonly referred to as coordinate descent (Friedman *et al.* 2010).

Consider the following univariate linear model fitting phenotypes as a function of an intercept and genotypic information:

y=1μ+Mβ+ϵ (1)

where *y* corresponds to a vector of phenotypes, *μ* is the intercept, *M* is a matrix of parameters where each $m_{ij}$ cell corresponds to $j^{th}$ locus of the $i^{th}$ individual coding {AA,Aa,aa} as {−1,0,1}, *β* refers to the vector of marker effects, *ϵ* represent the vector of residuals.

The first operation in each iteration is the intercept update as:

$$\mu =n^{-1}\sum_{i=1}^{n}\left(y_{i}-M_{i}\beta \right)$$ (2)

Marker effects and regularization parameters are updated one at a time until convergence. Conditioning the response to all but the $j^{th}$ marker ($\tilde{y}=y-1\mu -M_{-j}\beta_{-j}$) provides a simple probabilistic structure:

$$\tilde{y}|m,\beta ∼N\left(m_{j}\beta_{j},\sigma_{\epsilon }^{2}\right)$$ (3)

$$\beta_{j}|\tau_{j}^{2}∼N\left(0,\tau_{j}^{2}\sigma_{\epsilon }^{2}\right)$$ (4)

where $m_{j}$ is a vector containing the information of the $j^{th}$ marker, $\tau_{j}^{2}$ is the parameter that regularizes $\beta_{j}$, as the marker effect associated with the $j^{th}$ marker is estimated as:

$$\beta_{j}=\frac{m_{j}^{\prime }\tilde{y}}{m_{j}^{\prime }m_{j}+\tau_{j}^{-2}}$$ (5)

Each marker has an independent regularization. The regularization parameter $\tau_{j}^{-2}$, which shapes the marker effects collectively into a Laplace distribution, is derived from an inverse-Gaussian density with expectation (Park and Casella 2008):

$$\tau_{j}^{-2}=\sqrt{\lambda^{2}\sigma_{\epsilon }^{2}\sigma_{\beta_{j}}^{-2}}$$ (6)

The scale parameter $\lambda^{2}$ was adapted from Legarra *et al.* (2011), as the sum of marker variances:

$$\lambda^{2}=\sum_{j=1}^{p}\sigma_{m_{j}}^{2}$$ (7)

Residual variance and full-conditional marker variance are estimated by maximum likelihood (Patterson and Thompson 1971, Harville 1977, Searle *et al.* 1992):

$$\sigma_{\beta_{j}}^{2}=\frac{{\beta_{j}}^{\prime }\beta_{j}+tr\left({\tilde{C}}^{-1}\right)\sigma_{\epsilon }^{2}}{q}=\beta_{j}^{2}+\frac{\sigma_{\epsilon }^{2}}{m^{\prime }m+\tau_{j}^{-2}}$$ (8)

$$\sigma_{\epsilon }^{2}=\frac{y^{\prime }Py}{n-r_{X}}=\frac{y^{\prime }\epsilon }{n-r_{X}}$$ (9)

where *n* corresponds to the total number of observations, *q* is the number of parameters (q=1), ${\tilde{C}}^{-1}$ represents the inverse of the full-conditional left-hand side, *P* is the projection matrix of the whole model (Py=y−Hy=ϵ), and $r_{X}$ represents the rank of the design matrix of fixed effects ($r_{X}=1$).

The optimization path consists of iteratively updating *μ*, $\beta_{1}$, $\sigma_{\beta_{1}}^{2}$, $\tau_{\beta_{1}}^{-2}$, $\beta_{2}$, $\sigma_{\beta_{2}}^{2}$, $\tau_{\beta_{2}}^{-2}$, … and $\sigma_{\epsilon }^{2}$. The pseudo-code for the implementation is provided below (Algorithm 1) and an implementation for R is provided in the appendix. In this study, the convergence criteria was set as $10^{-8}$ for marker effects or a maximum of 300 iterations.

## Iterative single-stage method

The previous section presented how the algorithm for FLM works in the case where each individual has a single phenotypic value. Now consider the scenario of replicated trials, where genotyped individuals are replicated across multiple environments. This approach is here referred to as fast Laplace model in single-stage (FLM-SS). The term ”single-stage” has been used to define the joint modeling of replicated observations at the phenotypic level (Piepho *et al.* 2012) or with genomic information (Schulz-Streeck *et al.* 2013), which is not to be confused with the ”single-step” that elsewhere defines models that combine pedigree and genomic information (Misztal *et al.* 2009).

**Algorithm 1** Fast Laplace model

**Setup initial values:**

1. Compute $m_{j}^{\prime }m_{j}$ for each marker
2. Compute $\lambda^{2}=\sum_{j=1}^{p}\sigma_{m_{j}}^{2}$
3. Set $\lambda^{2}$ as initial value for all $\tau_{j}^{-2}$

**Repeat until convergence:**

1. Update intercept
  $$\mu^{t+1}=\mu^{t}+n^{-1}\sum_{i=1}^{n}\epsilon_{i}$$
  $$\epsilon^{t+1}=\epsilon^{t}-\left(\mu^{t+1}-\mu^{t}\right)$$
2. Loop for $j^{th}$ marker in 1:p
  $$\beta_{j}^{t+1}=\frac{m_{j}{}^{\prime }\epsilon^{t}+\beta_{j}^{t}\left(m_{j}{}^{\prime }m_{j}\right)}{m_{j}{}^{\prime }m_{j}+\tau_{j}^{-2}}$$
  $$\epsilon^{t+1}=e^{t}-m_{j}{}^{\prime }\left(\beta_{j}^{t+1}-\beta_{j}^{t}\right)$$
  $$\sigma_{\beta_{j}}^{2}=\beta_{j}^{2}+\frac{\sigma_{\epsilon }^{2}}{m_{j}{}^{\prime }m_{j}+\tau_{j}^{-2}}$$
  $$\tau_{j}^{-2}=\sqrt{\lambda^{2}\sigma_{\epsilon }^{2}\sigma_{\beta }^{-2}}$$
3. Update residual variance
  $$\sigma_{\epsilon }^{2}=\frac{y^{\prime }e}{n-1}$$

Most iterative methods provide an alternative way of fitting mixed model without changing its statistical properties. Efficient algorithms to solve complex models with marker information use conditional expectations, going back and forth with the estimation of fixed and random effect coefficients, without altering the outcome (Garrick 2007, Legarra and Misztal 2008, VanRaden 2008). The following model can illustrate the single-stage procedure:

y=Xb+Za+e (10)

where *y* is the vector of phenotypes, *X* and *b* represent the design matrix and fixed effect coefficients used to capture nuisance parameters, such as environmental sources of variation. The random terms *Z* and *a* correspond to the incidence matrix of individuals and additive genetic effects, hereby estimated from the WGR (a=Mβ). Residuals (e=y−Xb−Za) are assumed to be normally distributed as $e∼N\left(0,R\sigma_{e}^{2}\right)$, such that residuals can be autocorrelated (R≠I) or not (R=I).

Fixed effect coefficients are solved via least squares, conditioning the response variable to all terms but the fixed effect. This conditioning works by reshaping the linear model into:

y−Za=Xb+e (11)

Providing the following solution of coefficients:

$$b={\left(X^{\prime }R^{-1}X\right)}^{-1}X^{\prime }R^{-1}\left(y-Za\right)$$ (12)

To avoid building large and dense design matrix of marker effects (ZM), the random effect coefficients are updated using a link function in two steps ($u_{0}\to a$). First, estimate the least-squared genetic values ($u_{0}$) as follows:

$$y-Xb=Zu_{0}+e$$ (13)

The coefficients are solved as:

$$u_{0}={\left(Z^{\prime }R^{-1}Z\right)}^{-1}Z^{\prime }R^{-1}\left(y-Xb\right)$$ (14)

Note the values of $u_{0}$ are not the final random coefficients, but intermediary outcomes serving as input to WGR. The shrinkage of genetic values occurs when $u_{0}$ is updated into *a*.

Subsequently, the WGR algorithm introduced in the previous section takes place, solving the following equation to estimate marker effects and breeding values:

$$u_{0}=M\beta +\epsilon$$ (15)

In this case, the vector of residuals (***ϵ***) represents genetic signal not captured by the markers. Genotypes may have variable number of observations and weights can be assigned to the genotypes to account for the unbalancedness. The WGR step can be solved assuming unweighted observations for computational convenience, or weighted according to the number of observations of each genotype, thus $\omega =Diag\left(Z^{\prime }Z\right)$. The last step of the iteration regards updating the vector of breeding values as:

a=Mβ (16)

**Figure 1:**
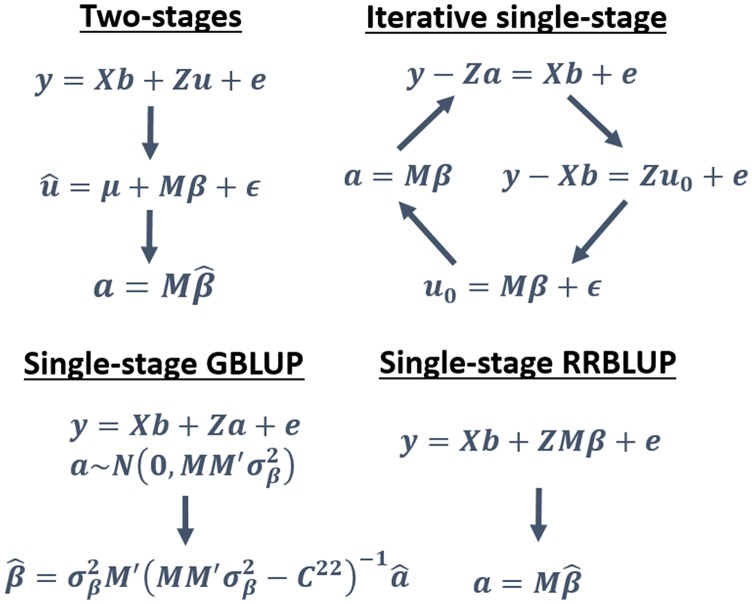
Approaches for modeling breeding values (*a*) and marker effects (*β*) from two-stages and different single-stage models.

In summary, this single-stage algorithm works through the iterative update of *b*, *a*, and *β* until convergence (Figure 1). Using Gauss-Seidel (Legarra and Misztal 2008) to update coefficients, this system of equations mitigates the computational burden of building and inverting large matrices. It must be acknowledged that the proposed algorithm is a computational trick and it may not replicate the same exact outcome of modeling the ZM matrix.

## Additional random effects

The study has focused on simple mixed models with fixed effects and a single random effect to model genetics. However, it often necessary to include random terms to capture genotype-environment interactions and spatial trends. This section describes how additional random effects can be included into the single-stage approach through conditioning.

Consider a model with one additional random effect:

y=Xb+Za+Wg+e (17)

Conditioning the response variable to all effects but the additional random effect ($\tilde{y}=y-Xb-Za$), yields:

$$\tilde{y}=Wg+e$$ (18)

Assuming $g∼N\left(0,I\sigma_{g}^{2}\right)$, the solution for the the random effect coefficients is given by:

$$g={\left(W^{\prime }R^{-1}W+kI\right)}^{-1}W^{\prime }R^{-1}\tilde{y}$$ (19)

where $k=\sigma_{e}^{2}\sigma_{g}^{-2}$. The solution for the residual variance is provided in equation (9) replacing *ϵ* by *e*.

Conditional to other model terms, the variance component associated to this random effect is estimated as (Patterson and Thompson 1971, Harville 1977):

$$\sigma_{g}^{2}=\frac{g^{\prime }g}{n_{w}-tr\left({\tilde{C}}^{-1}\right)k}=\frac{g^{\prime }g}{n_{w}-k\sum_{j=1}^{n_{w}}{\left({w_{j}}^{\prime }w_{j}+k\right)}^{-1}}$$ (20)

where $n_{w}$ is the number of columns of *W*, and $\tilde{C}$ is the full-conditional left hand-side equation. For random effects with non-orthogonal design matrices, such as adjacent matrices to model spatial auto-correlation, the variance component can be efficiently approximated as (Schaeffer 1986):

$$\sigma_{g}^{2}≅\frac{{\left(y-Xb\right)}^{\prime }Wg}{n\sum_{j=1}^{n_{w}}\sigma_{w_{j}}^{2}}$$ (21)

With more random terms in the model, all coefficients (*b*, *a*, and *β*) must be updated conditional to *g*.

## Materials and Methods

### Dataset

The soybean dataset described below was utilized to assess genomic prediction methodologies. The cross-validation analyses were performed on the genetic merit the genotypes estimated beforehand. These data were not used for the evaluation of the single-step procedure.

The soybean dataset contains 40 bi-parental families that share a common parent. Phenotypes and genotypes were obtained using the function ’BLUP’ from the R package SoyNAM. Each family contains approximately 140 individuals genotyped with 4320 markers, and the number of polymorphic markers ranged from 547 to 1262 within family. The soybean trait under evaluation was the best linear unbiased predictors (BLUP) of grain yield collected in as many as 18 environments under a modified augmented design (Lin and Poushinsky 1983). BLUPs were generated by modeling grain yield as a function of environment (random effect), genetic merit (random effect) and local block effects estimated from the checks (fixed effect). The population and experimental settings are described in details by Diers *et al.* (2018) and Xavier *et al.* (2018).

The BLUPs are not optimal for conducting second stage analyses (Smith *et al.* 2001), but were used for demonstration here, as raw plot level data were not available. BLUPs may create a heteroscedastic scenario that may or may not affect the prediction accuracy if not accounted for (de los Campos *et al.* 2013, Ou *et al.* 2016). This pitfall can be addressed by the deregression of coefficients (Garrick *et al.* 2009), where the genetic merit of individuals is unshrunken based on the individual’s reliability.

### Cross-validation

The cross-validation focused on two criteria: 1) the predictive ability measured as the correlation between predicted and observed genetic values, computed within-family from fivefold cross-validations with individuals were sampled at random (80% calibration, 20% validation) in each round and this procedure was repeated 20 times, and leave-family-out cross-validation by using 39 families to predict the family left out, and repeating this procedure for all 40 families; and 2) the elapsed time for calibrating the model using the whole data.

### Prediction methods

FLM was compared to a set of methods designed for high dimensional problems that are implemented and freely available in R. Including: Bayesian alphabet (A, B, C, RR, L) and reproducing kernel Hilbert spaces (RKHS) implemented in BGLR (Pérez and de Los Campos 2014); BayesC*π* and BayesD*π* implemented in the bWGR; GBLUP with REML variance components implemented in rrBLUP (Endelman 2011); boosting implemented in gbm (Ridgeway 2007); $L_{1}L_{2}$ machines - ridge regression, elastic-net and LASSO implemented in glmnet (Friedman *et al.* 2010); partial least square (PLS) implemented in pls (Mevik and Wehrens 2007); random forest implemented in ranger (Wright and Ziegler 2015); *ν* and *ϵ* support vector machines (SVM) implemented in kernlab (Karatzoglou *et al.* 2004); the empirical Bayesian LASSO (Cai *et al.* 2011) implemented in EBglmnet (Huang and Liu 2016); and the extended Bayesian LASSO (Legarra *et al.* 2011) implemented in VIGoR (Onogi and Iwata 2016). The latter two methods are efficient implementations with Laplace prior.

Methods above were deployed with default settings. Tuning parameters for ridge, LASSO and elastic-net were computed through 10-fold cross validation in the training set. To mitigate the computational burden necessary to tune parameters, PLS used 5 components and the empirical Bayes Lasso hyperparameters a-b were set to 0.5. The Gaussian kernel employed for RKHS was computed as $K=exp\left(-\delta D^{2}\right)$ where $D^{2}$ is the squared Euclidean distance matrix computed from the marker information and *δ* is the average value of $D^{2}$. The GBLUP model utilized the genomic relationship matrix described by VanRaden (2008).

### Detection of QTL

An experimental population was generated through simulation to evaluate FLM estimates of large effect parameters. From an F2 bi-parental cross with 1000 individuals, 250 individuals were randomly selected and randomly mated to generate a new population of 1000 individuals. This bottle-necking with subsequent random mating was repeated 5 times. The resulting allele frequency ranged from 0.32 to 0.63. The simulated genome had 10 chromosomes of length 100 cM. The genotyping density was 0.5 marker/cM. A causative marker was assigned to the center of each chromosome with alternating values of positive and negative one.

The response variable was evaluated under heritability of 0.25 and 0.50. The ability of FLM to detect major genes was compared to the Bayesian ridge regression and Bayesian LASSO implemented in the R package BGLR (Pérez and de Los Campos 2014), and a mixed model association based on P3D algorithm (Zhang *et al.* 2010) implemented in the R package NAM (Xavier *et al.* 2015). Three population sizes were evaluated to estimate the allele effects: 250, 500 and 1000 individuals.

### Evaluation of single-stage method

Breeding data are inherently unbalanced. Genotypes are often unreplicated or not equally distributed across environments, and observations from different environments present a variable degree of noise. The single-stage approach was evaluated on simulated datasets that recreated such condition.

#### Simulated dataset:

The simulations were based on assigning the simulated individuals described in the previous section, a genetic pool with 1000 genotypes, to a random set of environments. Each simulated scenario was performed with a combination of number of observations across trials (n = 250, 500, 1000, 2500, 5000, 10000) and genetic architectures (10, 50 and 100 QTL). The number of environments for each simulation was sampled from a uniform distribution between 4 and 10 with effect N(μ=100,σ=20). To simulate heteroscedasticity, each environment had a different heritability sampled from a uniform distribution between 0.25 to 0.75. Individuals were sampled with replacement, such that each environment had an unequal number of entries. Each scenario (combination of size and genetic architecture) was repeated 20x with different seeds to sample the individuals, number of locations, and heritability of the locations.

For the simulated scenarios with less than 1000 observations, most genotypes were unreplicated since the observed individuals were sampled from a pool of 1000 genotypes. Selection across unreplicated trials are not unusual when genomic prediction is deployed (Sebastian *et al.* 2010) since genotypes are connected through the relationship information captured by markers (Habier *et al.* 2007). Phenotypic values were generated by adding an environmental effect and random noise to the true breeding values. For simplicity, genotype-by-environment interactions, non-additive genetics, and spatial noise were not considered.

#### Prediction methods:

Three methods were evaluated. 1) Iterative single-stage with Laplace (FLM-SS) and Gaussian prior (ridge regression, RR-SS), implemented in RcppEigen (Eddelbuettel *et al.* 2011). 2) Two-stage approach described by Schulz-Streeck *et al.* (2013) based on fitting the best linear unbiased estimators (BLUE) of genetic values without using genomic information (first step), treating environment as a random effect, and subsequently fitting a WGR (second step) to estimate breeding values. The first-stage BLUEs were computed with the lme4 package (Bates *et al.* 2015), and markers were fitted with the Bayesian LASSO implemented the BGLR package (Pérez and de Los Campos 2014), carrying over the covariances from the first-stage to account for the environmental heteroscedasticity, assuming the second-stage residual covariances to be inherited from the first-stage. 3) GBLUP fitted with the commercial software ASReml (Gilmour *et al.* 2008) using a genomic additive relationship matrix (Zeng *et al.* 2005, Xu 2013). GBLUP is also a single-stage procedure to generate breeding values (Figure 1), however marker effects are not explicitly computed for the prediction on new individuals.

#### Evaluation criteria:

The criteria for comparison was the computation time necessary to fit the model as the elapsed time, and the prediction accuracy as the correlation between estimated breeding values and true breeding values.

#### Statistical models:

The evaluated models aim to estimate breeding values (a=Mβ) from phenotypes (*y*). GBLUP and Single-Stage fit environment (Xb) as fixed effect and genetics as random effect, as Za and Z(Mβ), respectively. The two-stages fits environment (Xb) as random and genetic merit as fixed effect (Zu) in the first stage, followed by modeling the genetic merit (*u*) as function of intercept (*μ*) and marker effects (Mβ), weighting observations with the coefficient variance, $S=Diag\left(Z^{\prime }V^{-1}Z\right)$, where $V=XX^{\prime }\sigma_{b}^{2}+I\sigma_{e}^{2}$, which translates into observations with weights $S^{-1}$.

The three models can be summarized as follows:

1. Iterative single-stage (FLM-SS, RR-SS):
  $$\begin{array}{c} y=Xb+Z\left(M\beta \right)+e \end{array}$$ (22)
2. Two-stage approach:
  $$\begin{array}{c} y=Xb+Zu+e\\ u=\mu +M\beta +\epsilon ,\;\epsilon ∼N\left(0,S\sigma_{\epsilon }^{2}\right) \end{array}$$ (23)
3. GBLUP:
  $$\begin{array}{c} y=Xb+Za+e,\;a∼N\left(0,MM^{\prime }\sigma_{\beta }^{2}\right) \end{array}$$ (24)

### Data availability

The soybean data are available in the R package SoyNAM. The R code for FLM is in the appendix. Cross-validation scripts are available on GitHub (*github.com/alenxav/FLM*). The FLM-SS code can be made available for research purposes.

## Results

### Genomic prediction analysis

**Figure 2:**
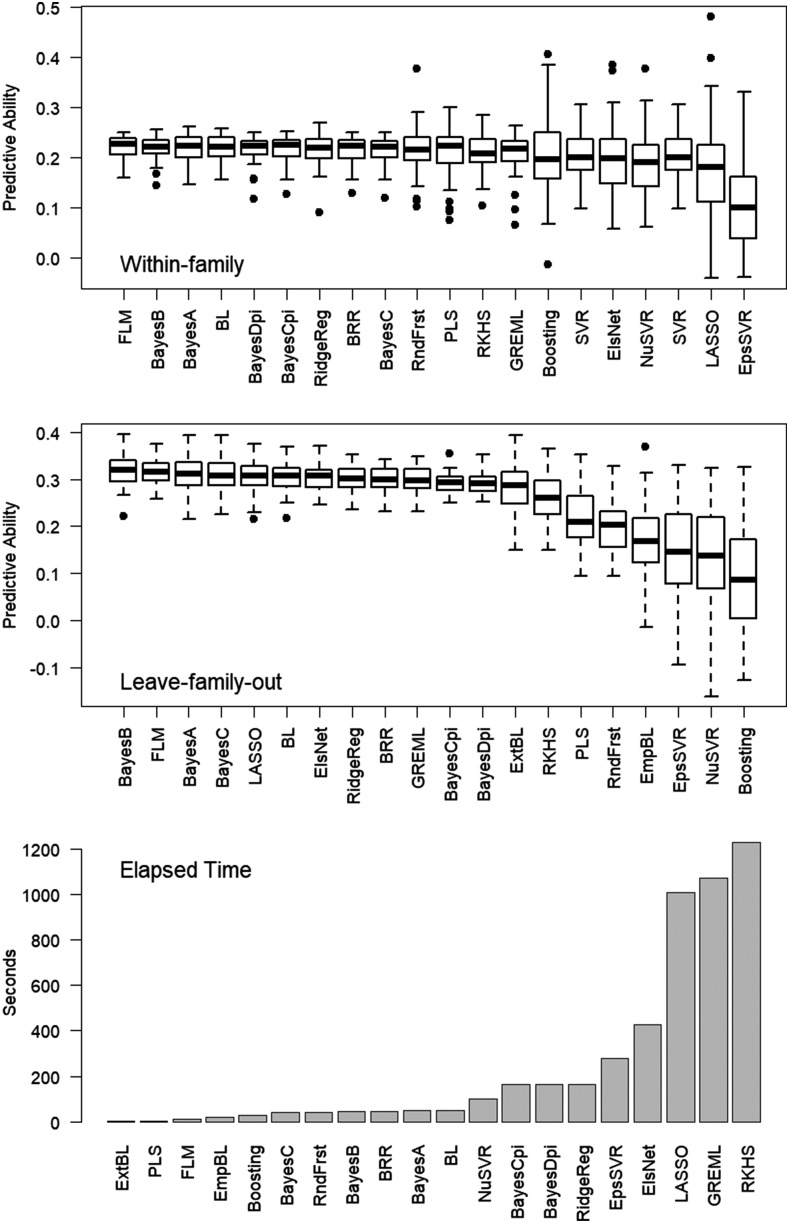
Box-and-whiskers plot displaying the predictive ability across 40 bi-parental soybeans families. Prediction within-family (top), leave-family-out (center) and computation time (bottom).

The summary of prediction statistics from cross-validation is presented in Figure 2. FLM was the most predictive methodology within-family and the second most predictive under leave-family-out cross-validation. Most methods provided satisfying predictive ability. Considering computation time (Figure 2), PLS and the three non-MCMC implementations of the Laplace prior had the lowest computational cost. Kernel methods had high computational cost.

### Learning properties

**Figure 3:**
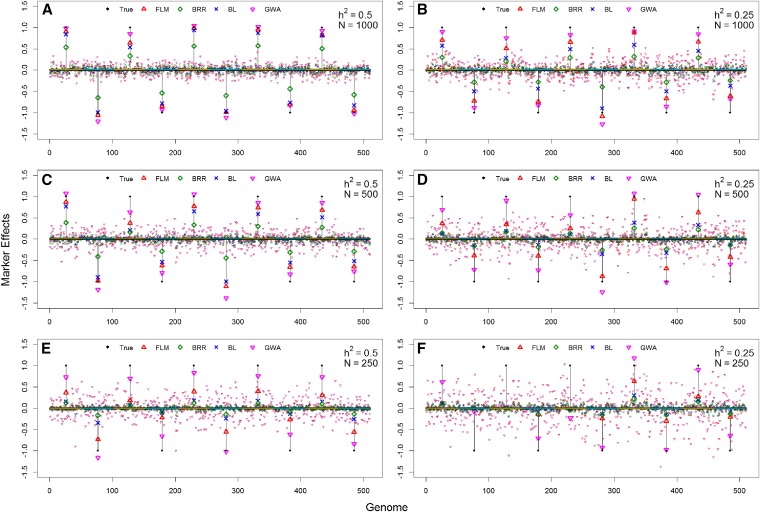
Simulation-based evaluation of marker effect estimation (y-axis) across the genome (x-axis) with varying the heritability and number of individuals, testing: Fast Laplace model (FLM), Bayesian ridge regression (BRR), Bayesian LASSO (BL), genome-wide associations (GWA) analysis, and the true value (True). Effects were plotted larger at the QTL positions and smaller in every other locus.

The ability of different approaches to correctly estimate major effects through simulation is presented in Figure 3. Marker effects estimated from genome-wide association analysis were the closest to the true simulated values, however GWA resulted in an abundance of false signals across the genome as the marker effects are not estimated conditional to the neighbor markers.

The allele effect estimated by FLM was closer to the true value than its MCMC counterpart, the Bayesian LASSO, and this difference was more evident in the low heritability scenario (Figure 3 bdf). Bayesian ridge regression captured the large effects reasonably well in scenarios with where the heritability was 0.5, but the estimates were not close to the real values in any situation. In general, more realistic values were achieved by all methods as the population size and heritability increased.

### Single-stage efficiency

**Figure 4:**
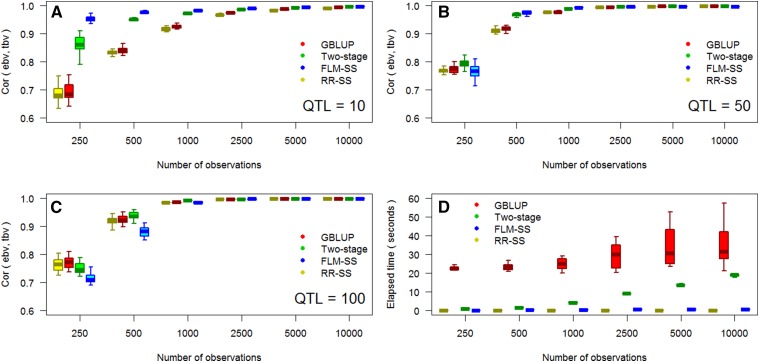
Simulation-based comparison of methods: GBLUP from replicated trials (GBLUP, red); two-stages approach where whole-genome regression fitted on genetic values (Two-stage, green); and an iterative single-stage approach, FLM single-stage (FLM-SS, blue) and Ridge Regression single-stage (RR-SS, yellow). Accuracy under 10 QTL (a), 50 (b) and 100 (c) QTL and elapsed time (d) to fit the model.

The comparison of accuracy and speed among GBLUP, two-stages approach, and iterative single-stage (FLM-SS, RR-SS) is presented in Figure 4. As the number of observations increased, all methods converged to the simulated true values. GBLUP was slightly better than its iterative single-stage counterpart, RR-SS, indicating that the proposed algorithm provides a comparable, however not identical, predictive outcome. This small difference is likely due to how well methods handle information unbalancedness.

The accuracy of Gaussian prior models (GBLUP and RR-SS) was sensitive to the number of observations when the trait was controlled by a small number of QTL. As the number of QTL increased, the predictive advantage of FLM-SS and two-stages over GBLUP and RR-SS decreased, and GBLUP outperformed the other methods under the scenario with the lowest number of observations.

The computation time of GBLUP was considerably larger than iterative single-stage, and two-stage approach yielded intermediate computational performance. The discrepancy in computation time between iterative single-stage and two-stages can be attributed to the MCMC sampling in the second step and the estimation of variance components in the first step.

## Discussion

The discussion section frames FLM as a potential method of choice for genomic prediction in plant breeding. The proposed methodology provided accurate prediction across datasets, as well as computational efficiency. Besides the predictive and computational performance, the FLM is an easy-to-implement regression method (Algorithm 1) without the need for tuning or matrix inversion.

### Predictive ability

Most methods provide comparable predictive performance (Pérez-Rodríguez *et al.* 2012, Howard *et al.* 2014, Xavier *et al.* 2016). This study compared prediction methods within-family and family-out predictions, with predictive ability around 0.3 and 0.5, respectively, consistent with literature (Legarra *et al.* 2008, Lian *et al.* 2014, Xavier *et al.* 2016). Within-family predictions rely on modeling the Mendelian segregation between markers and QTL, whereas across-family predictions are based on capturing the relationship among families (Habier *et al.* 2007, Daetwyler *et al.* 2013, Lehermeier *et al.* 2014). FLM provided competitive values of predictive ability for both validation methods. However, the predictive performance of models may vary according to genetic architecture, marker density, trait heritability, and the size of the training set (de los Campos *et al.* 2013, Legarra *et al.* 2015).

Feature selection is a desirable statistical property known to improve the parsimony and predictive ability of WGR models (Wimmer *et al.* 2013). FLM deploys the so-called Laplacian variable selection (O’Hara and Sillanpää 2009), which imposes strong shrinkage without eliminating the parameters from the model. Markers not linked to QTL often play an important role on prediction by capturing relationship among individuals (Habier *et al.* 2007). In addition, when regression coefficients have priors shaped by heavy tailed distribution, such as Laplace and Student’s t, models are suited to capturing QTL because these priors relax the shrinkage of markers with large effect (de los Campos *et al.* 2009, Kärkkäinen and Sillanpää 2012). Other models with similar properties include BayesA, BayesB, BayesC and the Bayesian LASSO (de los Campos *et al.* 2009, Habier *et al.* 2011, Heslot *et al.* 2012, Kärkkäinen and Sillanpää 2012, Legarra *et al.* 2015).

From the signal detection perspective, models able to capture relationship and accurately detect QTL are deployed for association studies and haplotype analysis (Fernando and Garrick 2013, Hayes 2013, Yang *et al.* 2014, Daetwyler *et al.* 2015, Fernando *et al.* 2017, Goiffon *et al.* 2017). For the scenarios under evaluation, FLM provided a more accurate marker effects estimation than the Bayesian LASSO and ridge regression, with less spurious association than GWA (Figure 4). Both FLM and Bayesian LASSO have a Laplace prior, but with substantial algorithmic differences. Empirical priors have been reported to improve the predictive properties of Laplace models (Xu 2007, Yi and Xu 2008, Xu 2010, Cai *et al.* 2011), thus FLM likely benefits from regularization free of hyperparameters. Moreover, iterative algorithms often outperform their MCMC counterpart in terms of accuracy (Hayashi and Iwata 2010, Sun *et al.* 2012, Wang *et al.* 2015). The resulting improvement in signal detection translates into higher predictive ability in scenarios where capturing linkage disequilibrium is more important than the relationship among individuals, as depicted in within-family predictions (Figure 2).

The genetic signal captured by WGR methods is solely additive, which is desirable to estimate breeding values but sub-optimal for the prediction of phenotypes. Unlike additive models, semi-parametric methods can capture non-linear relationship patterns and different levels of epistasis. For this reason, additive models are frequently outperformed by semi-parametric methods, such as RKHS, SVR, random forest and neural networks (Gianola *et al.* 2006, De Los Campos *et al.* 2010, Pérez-Rodríguez *et al.* 2012, Desta and Ortiz 2014, Howard *et al.* 2014). For the datasets under evaluation, linear models were as predictive as semi-parametric methods, which suggests that most genetic signal was due to additive genetics.

Both RKHS and *ϵ*-SVR are kernel methods that utilize a Gaussian kernel, but these methods differ with regards to their loss-functions. Whereas RKHS follows a $L_{2}$ loss that penalizes square error and coefficients, *ϵ*-SVR only penalizes the error greater than *ϵ* (Hastie *et al.* 2009). Interestingly, *ν*-SVR did not provide the same degree of predictive ability, despite sharing the same kernel as RKHS and *ϵ*-SVR.

### Computational performance

The time required to calibrate a prediction machine is an important factor to chose a methodology when genomic prediction is utilized for various traits, with often model re-calibration (Meuwissen *et al.* 2009, Hayashi and Iwata 2010, Sun *et al.* 2012, Wang *et al.* 2015). Results indicate a clear discrepancy across methods with regards to the computing time required to fit the prediction models. Figure 2 shows the most computationally efficient methods were PLS and the non-MCMC implementations. Other regression-type methods provided intermediate efficiency and kernel-type methods were computationally expensive.

Most prediction methods display some computation burden: tuning parameters in machine learning methods; MCMC iterations in Bayesian methods; variance components in GBLUP; and matrix inversion or decomposition in kernel methods. FLM estimates full-conditional variance components, dismissing expensive matrix operations, cross-validation for tuning parameters, or MCMC. The other two prediction methods that efficiently implement Laplace prior, the empirical Bayesian LASSO and the extended Bayesian LASSO, did not provide satisfactory predictive ability. Besides FLM, our results indicate that BayesB is also a cost-effective regression method by providing reasonable computational cost with hist predictive ability across datasets.

It is important to point out that kernel methods can be a suitable alternative in high dimensional models, since these rely on the number of individuals rather than the number of parameters. Kernel methods are computationally demanding for two other reasons: it is necessary to 1) build the kernel and 2) compute its inversion or Eigendecomposition. The time needed to build the kernel depends on the number of both individuals and parameters. Many kernels require the computation of distance matrices, which is more computationally demanding.

For the prediction of new observations, kernels must be augmented with the genotypes of observed and unobserved individuals, making the inversion or spectral decomposition more challenging. This is particularly cumbersome in plant breeding where the size of the offspring being predicted and selected can be much larger than the training set, whereas the parameter estimates required for prediction from regression and tree models can be stored and easily employed for prediction of new observations.

### Iterative single-stage modeling

The two-stage and iterative single-stage approaches were faster than GBLUP by an order of magnitude. Such difference can be attributed to the sparse nature of the algorithm and the complexity associated to the estimation of variance components. GBLUP was fit using AI-REML, a general-purpose algorithm, whereas FLM-SS was specifically designed to provide efficient computation of breeding models. The two-stages model provided an intermediate outcome.

The lower accuracy of the GBLUP and RR-SS in scenarios with few QTL can be attributed to the statistical nature based an infinitesimal model. Gaussian priors work by capturing the relationship among individuals (Habier *et al.* 2007), whereas FLM-SS and two-steps models enable fitting priors that are suitable to capture both relationship and QTL (Wolc *et al.* 2016). Similar results were reported by Zhou *et al.* (2018), where one-step BayesA and BayesB consistently outperformed the one-step GBLUP under various simulated scenarios. This advantage is also depicted in within-family prediction (Figure 2) where the prediction power comes from detecting LD between markers and QTL, as well as in Figure 3, where it takes a larger number of observations to BRR (counterpart of GBLUP) to identify large effect markers (de los Campos *et al.* 2013, Henryon *et al.* 2014, Hickey *et al.* 2017).

Frameworks where marker effects are estimate alongside all other parameters are not new, but rarely utilized (Fernando *et al.* 2014, Liu *et al.* 2014, Taskinen *et al.* 2017). Methods and implementations of genomic prediction have been incorporated from animal breeding into plant breeding without much consideration about the large differences in data flow and other statistical properties (Heslot *et al.* 2015, Hickey *et al.* 2017). Two of the major factors that differentiate plant and animal breeding are replicated trials and offspring size. The single-stage framework proposed in this study was design for genomic prediction following the plant breeding data structure, being beneficial from the computational and predictive standpoint.

### Two-stages and iterative single-stage

Multi-stage procedures have been proposed to address the computational burden of single-stage without compromising in the quality of the results (Smith *et al.* 2001). There are various methods to propagate weights from one stage to the next (Möhring and Piepho 2009). However, multi-stage analysis cannot recreate the exact results of single-stage unless the whole covariance matrix is carried over stages (Piepho *et al.* 2012). Even so, the results of multi-stage only reproduce single-stage if the variance components of the first-stage analysis are identical to the variance components estimated from single-stage, and if the nuisance parameters (non-target fixed effects) are estimated free of error. In this case, sufficient statistics (means and covariance) may fully reproduce single-stage. Multi-stage approaches commonly provide results comparable to those from single-stage analysis (Damesa *et al.* 2017). However, if statistical terms introduced in the second-stage analysis would potentially affect the variance component estimates of first-stage terms, then the single-stage results cannot be exactly reproduced by two-stages. That is not necessarily the case for iterative methods where all coefficients and the variance components are estimated conditional to each other, and the information of all terms is propagated across iterations.

Future directions for this research include the comparison of breeding values estimated from GBLUP, multi-stage and RR-SS to understand how the proposed algorithm reproduces standard procedures on real data. An evaluation of the estimated variance components is also a desirable target. Further studies must contrast scenarios with genotype-environment interaction, spatial trends, dominance and epistasis.

### CONCLUSION

A robust prediction methodology is a key component for a successful genomic-assisted breeding pipeline. This study introduced a fast and accurate algorithm for solving a WGR with Laplace prior, alongside a single-stage methodology that allows to connect WGR into mixed models with replicated observations.

The proposed framework provided more accurate predictions and higher computational efficiency than other methods based on a cross-validation evaluation on real datasets. With a simulated dataset, it was shown that the fast Laplace model provided reasonably accurate estimation of QTL effects, being less biased than Bayesian LASSO and ridge regression, and proving less spurious signals than genome-wide association analysis. The algorithm extension to single-stage also presented promising properties, benefiting both computation and prediction.
